# Artificial Neural Network Genetic Algorithm As Powerful Tool to Predict and Optimize *In vitro* Proliferation Mineral Medium for G × N15 Rootstock

**DOI:** 10.3389/fpls.2016.01526

**Published:** 2016-10-19

**Authors:** Mohammad M. Arab, Abbas Yadollahi, Abdolali Shojaeiyan, Hamed Ahmadi

**Affiliations:** ^1^Department of Horticultural Science, Faculty of Agriculture, Tarbiat Modares UniversityTehran, Iran; ^2^Department of Horticultural Sciences, College of Abooraihan, University of TehranTehran, Iran; ^3^Department of Poultry Sciences, Faculty of Agriculture, Tarbiat Modares UniversityTehran, Iran

**Keywords:** artificial neural network (ANN), genetic algorithm (GA), G × N15 rootstock, ion macronutrients, proliferation, *Prunus* micropropagation

## Abstract

One of the major obstacles to the micropropagation of *Prunus* rootstocks has, up until now, been the lack of a suitable tissue culture medium. Therefore, reformulation of culture media or modification of the mineral content might be a breakthrough to improve *in vitro* multiplication of G × N15 (garnem). We found artificial neural network in combination of genetic algorithm (ANN-GA) as a very precise and powerful modeling system for optimizing the culture medium, So that modeling the effects of MS mineral salts (${\text{NH}}_{4}^{+}$, ${\text{NO}}_{3}^{-}$, ${\text{PO}}_{4}^{2-}$, Ca^2+^, K^+^, ${\text{SO}}_{4}^{2-}$, Mg^2+^, and Cl^−^) on *in vitro* multiplication parameters (the number of microshoots per explant, average length of microshoots, weight of calluses derived from the base of stem explants, and quality index of plantlets) of G × N15. Showed high *R*^2^ correlation values of 87, 91, 87, and 74 between observed and predicted values were found for these four growth parameters, respectively. According to the ANN-GA results, among the input variables, ${\text{NH}}_{4}^{+}$ and ${\text{NO}}_{3}^{-}$ had the highest values of VSR in data set for the parameters studied. The ANN-GA showed that the best proliferation rate was obtained from medium containing (mM) 27.5 ${\text{NO}}_{3}^{-}$, 14 ${\text{NH}}_{4}^{+}$, 5 Ca^2+^, 25.9 K^+^, 0.7 Mg^2+^, 1.1 ${\text{PO}}_{4}^{2-}$, 4.7 ${\text{SO}}_{4}^{2-}$, and 0.96 Cl^−^. The performance of the medium optimized by ANN-GA, denoted as YAS (Yadollahi, Arab and Shojaeiyan), was compared to that of standard growth media for all *Prunus* rootstock, including the Murashige and Skoog (MS) medium, (specific media) EM, Quoirin and Lepoivre (QL) medium, and woody plant medium (WPM) *Prunus*. With respect to shoot length, shoot number per cultured explant and productivity (number of microshoots × length of microshoots), YAS was found to be superior to other media for *in vitro* multiplication of G × N15 rootstocks. In addition, our results indicated that by using ANN-GA, we were able to determine a suitable culture medium formulation to achieve the best *in vitro* productivity.

## Introduction

The use of stone fruit inter-specific hybrids has been common in developed countries for a few decades, and this approach would improve many problems in stone fruit trees (Beckman and Lang, 2002). Improved outcomes, however, have been observed for the rootstock (A plant which already has an established root system, onto which scion is grafted) G × N15, also called “Garnem,” which is a hybrid between the stone fruits almond and peach selected from the plants originated by crossing *Prunus amygdalus* (Garfi) × *Prunus. Persica* (Nemared) (Series GxN), and which was developed in Spain at the Center of Investigation and Technology Agrifood of Arago (Felipe, 2009). This new rootstock is characterized by red leaves, good vigor, resistance to root-knot nematodes, adaptability to calcareous soils, and compatibility with most peach and almond cultivars as well as some grafted plum and apricot cultivars. Garnem also performs very well with almond, both in irrigated and rainfed (Felipe, 2009). Obtaining G × N15 hybrid and making their cloning is difficult. Therefore, optimize a new *in vitro* multiplication protocol for large-scale production of this rootstock is necessary.

The success and commercial usefulness of *Prunus* rootstock micropropagation protocols largely depend on the mode and rate of shoot proliferation (Number of new formed shoots). Shoot proliferation is influenced by several factors, such as genotype, media composition (Ruzic and Vujovic, 2008; Ivanova and Van Staden, 2009; Yang et al., 2012), disinfection and establishment methods (Shokri et al., 2013; Arab et al., 2014), *in vitro* environmental factors, etc. Development of an appropriate culture medium for a specific crop can be quite complex because the response to the culture medium is often genotype dependent, and the effects of mineral nutrition on morphogenesis have hardly been studied (Ramage and Williams, 2002; George et al., 2008; Greenway et al., 2012; Wada et al., 2013). Mineral nutrients include critical molecules of plant cells or function as critical parts of the cell structure (Ramage and Williams, 2002). Choosing the most appropriate medium culture in this regard is a vital but often overlooked consideration in many tissue culture applications (Greenway et al., 2012). Basal salts, for example, regulate the growth and morphology of plant tissues by providing essential nutrients (Ramage and Williams, 2002). Different species have different nutrient requirements that provide the optimal growth; both deficiencies and excesses of specific nutrients can result in negative impacts and physiological problems, such as vitrification (Ramage and Williams, 2002; Ivanova and Van Staden, 2009; Yang et al., 2012). These differences have led to the development of many medium formulations, but selecting a medium for a particular objective can be difficult and time consuming. Medium selection is often based on previous applications with many plant species. However, a basal culture medium selected based upon past usage may not give consistent results when evaluated with different plant species, different plant tissues or new applications (Greenway et al., 2012).

Currently, there are few media formulations that are broadly applicable for the micropropagation of *Prunus*. In some situations, the nutrient concentrations of these common media are slightly modified to accommodate a particular use (Nezami Alanagh et al., 2014). Murashige and Skoog (MS) (Murashige and Skoog, 1962) and woody plant medium (WPM) are the two most commonly used basal media, but they are not suitable for all *in vitro* applications (Nezami Alanagh et al., 2014). The MS, WPM, and Quoirin and Lepoivre (QL) basal salt formulations were not optimized for *in vitro* proliferation of G × N15 but became widely used because many other *Prunus* species grew well on them (Arab, unpublished data). Each plant species has its own characteristic requirement for nutrients, which can be used to adapt the medium formulation (Nas and Read, 2004; Ruzic and Vujovic, 2008; Radmann et al., 2009; Unek et al., 2010; Zhou et al., 2010; Pérez-Jiménez et al., 2012; Vujović et al., 2012; Jamshidi et al., 2016).

Micropropagation has been evaluated as an alternative to conventional vegetative propagation of *Prunus* rootstock by several research groups (Cheong, 2012), but standardized *in vitro* proliferation protocols for G × N15 rootstock have not yet been developed. Although mineral nutrition is one of the crucial factors for successful *Prunus* micropropagation, the effects of mineral nutrition used on *in vitro* G × N15 rootstock shoot regeneration from a single node has been investigated in only a few cases.

Utilization of artificial neural network (ANN) technologies and genetic algorithms (GAs) can be efficient alternative means for non-linear multivariate modeling and optimization of biological processes (Prasad and Dutta Gupta, 2006; Jong, 2009). Neural network technology is considered to be an alternative to the polynomial regression method for approximating of different complex mathematical functions to process and interpret many sets of unpredictable data (Ahmadi and Golian, 2011). ANN technology has been found to be completely applicable for experiments with different numbers of data points, which makes it possible to use more casual experimental designs than is allowed with statistical approaches (Ahmadi and Golian, 2011). Recently, several studies have demonstrated the effectiveness of ANNs in the field of plant tissue culture for different purposes such as predicting the number of shoots per explant and average shoot length (Gago et al., 2010a, 2011; Nezami Alanagh et al., 2014), modeling the weight of root biomass (Mehrotra et al., 2008, 2013; Prakash et al., 2010), and predicting the number of roots per microshoots and survival percentage (Gago et al., 2010a,b). ANN-GA is a hybrid technology that combines the adaptive learning capabilities from ANN with a GA. This methodology has proven its applicability in successfully modeling complex non-linear relationships between variables (Ahmadi and Golian, 2011). A genetic algorithm was used by these authors to optimize input space of an ANN model since GAs are often used as artificial intelligence based stochastic optimization methods for optimizing the input space of an ANN model where the usual methods are not applicable. The combination of ANN and GA techniques has become one of the most efficient methods used for experimental modeling and for selecting the best performers or optimization, especially to increase knowledge about the factors that potentially affect any responses in complex non-linear relationships hidden in formulation data (Ahmadi and Golian, 2011). The ANN-GA method has helped researchers understand cause–effect relationships, for example, between the composition of the culture medium and the growth parameters (i.e., number of new shoots formed, shoot length, quality, etc.).

*In vitro* proliferation of G × N15 on MS medium (1962) often exhibits physiological disorders such as hyperhydricity, shoot tip necrosis, discoloration, callus formation, leaf spots, fasciation, or stunting (Arab, unpublished data). In contrast, shoots grown on WPM are healthy but show a slow growth rate. Whereas, MS media resulted in high proliferation rate and plantlets often exhibit suboptimal growth and symptoms in this media (Arab, unpublished data). Finally, optimizing culture medium to provide satisfactory mineral nutrition for *in vitro* proliferation of new cultivars, such as, G × N15 is very challenging because each cultivar has its own specific nutritional requirements. The complexity of understanding how shoots respond to mineral nutrients in a medium can make developing an optimal medium very difficult and time consuming, as shown by the classic study of Murashige and Skoog in 1962 (Ramage and Williams, 2002). Recently, many new approaches have been developed for improving medium formulation based on mineral nutrition and better understanding the role of the factors involved in *in vitro* plant growth using kernel composition (Nas and Read, 2004; Adelberg et al., 2010) and computer technologies, especially ANNs (Gago et al., 2010b, 2011; Greenway et al., 2012; Mehrotra et al., 2013; Zielinska and Kepczynska, 2013; Nezami Alanagh et al., 2014; Poothong and Reed, 2014).

Our current study was designed to determine the mineral factors that have the greatest effects on the growth and development of G × N15 rootstock by using ANN-GA. The mineral salts of the WPM and QL media and MS base medium were used as a starting point for optimization of five mineral stock solutions for improved growth and multiplication of G × N15. The purposes of the present study were to: (1) develop ANN-GA models to analyze the response of G × N15 microshoots to mineral medium according to the number of new shoots formed and shoot length obtained from two factorial experiments based on a completely randomized design; (2) find the optimal culture medium composition for maximizing the number of proliferated shoots per explant, maximizing the average shoot length, maximizing the quality of the shoots, and minimizing calluses derived from basal microshoots by optimizing the model of G × N15 microshoots at the *in vitro* proliferation stage; (3) compare new media formulated by ANN-GA (YAS) with MS, WPM, EM (specific media), and QL in order to assess the efficiency of ANN-GA for modeling and optimizing the composition of the culture medium for growth parameters (i.e., number of proliferated shoots per explant, average shoot length, calluses derived from basal microshoots, and quality of the shoots); and (4) investigate the role of macronutrients in the culture media involved in the *in vitro* growth of G × N15 hybrid rootstock.

According to the literature review, all researchers believe that each plant species has a specific culture media for different stage of its micropropagation and the ANN-GA method could be applied as an alternative method for optimizing a new culture media. Therefore, the hypothesis of this study is that mineral nutrition affects plants performances and the obtained data of these experiments can be analyzed using alternatives procedures like ANN-GA.

## Materials and methods

The experiments were carried out in the Fruit Tree Micropropagation Laboratory, Department of Horticultural Sciences, Tarbiat Modares University (TMU) Tehran, Iran, in 2013 and 2014.

### Plant material

Cultures were initiated using nodal explants taken from actively growing approximately 2-year-old peach × almond hybrid rootstock G × N15 grown in a greenhouse at 24–26°C and a 16/8-h (light/dark) photoperiod at TMU. Nodal segments of lengths of 1–2 cm, including axillary buds, were used as explants. Single-node explants of the *Prunus* rootstock “Garnem” were taken in the spring of 2013 from trees propagated either by cuttings or by micropropagation. Actively growing axillary shoots (15–20 cm) of “Garnem” were cut off and transferred to the Fruit Tree Micropropagation Laboratory and used to prepare nodal cuttings. These shoots were cut into pieces that were 2 cm long, with each piece including one bud; then for surface disinfection, nodal explants were agitated in a solution containing water, liquid hand soap and 0.03% (v/v) Tween 20 (Merck, La Jolla, USA) for 15 min; and finally explants were washed with running tap water for 1 h. These explants were subjected to internal sterilization by immersing them in 70% (v/v) ethanol (Sigma-Aldrich, Italy) for 30 s, rinsing them in sterile distilled water and then submerging them for 4 min in mercury chloride (0.01%), with constant shaking. Later, the cuttings were immersed twice in double-distilled water containing 700 mg/L citric acid, each time for 3 min, and finally they were rinsed twice with distilled water, before transferring them to test tubes containing 15 mL of MS medium.

### *In vitro* culture establishment

After disinfection, MS medium (Murashige and Skoog medium, 1962) containing 0.25 mg L^−1^ BAP, 0.05 mg L^−1^ IBA (Sigma-Aldrich, Steinheim, Germany) and 30 g L^−1^ (Duchefa) sucrose was used as the culture medium for shoot induction. The pH of the culture medium used was adjusted to 5.8 with 0.1 M NaOH, and then 7.0 g L^−1^ agar (Merck microbiological) was added, and the medium was autoclaved at a temperature of 121°C and a pressure of 1.2 kPa for 15 min. Nodal explants (15–20 mm) were vertically cultured in 15 × 250-mm glass test tubes containing 10–15 mL of medium. Cultures were then subjected to a 16/8-h (light/dark) photoperiod at a light intensity of 80 μmol m^−2^ s^−1^ provided by white fluorescent tubes in a growth chamber for 4 weeks. One month after establishment of the culture, shoots that originated from the explants were sub-cultured on MS medium supplemented with 1 mg L^−1^ BAP and 0.1 mg L^−1^ IBA in a glass jar kept in a growth chamber.

### Preparation of media

Seventy six culture media were employed: MS (Murashige and Skoog medium, 1962), WPM (Lloyd and McCown, 1981), EM, QL (Quoirin and Lepoivre, 1977), modified MS predicted and optimized according to ANN-GA, modified MS (first experiment, 36) and modified MS and WPM (second experiment, 36) (Tables 1, 2). All media were supplemented with 1 mg L^−1^ BAP, 0.1 mg L^−1^ IBA, and 30 g L^−1^ sucrose. After adjustment of the pH to 5.7, 7.0 g L^−1^ agar (Merck microbiological) was added to the media. The media were prepared in 25 × 200-mm glass culture tubes. After autoclaving at 121°C and a pressure of 1.2 kPa for 15 min, the media were cooled to 65°C in a water bath, and then distributed into glass baby food jars (250 mL), each containing 50 mL of one type of medium, and the jars were closed with polypropylene screw caps. Before starting three sets of experiments to evaluate the effects of the various media, microshoots of G × N15 that were previously sub-cultured twice on MS containing 1 mg L^−1^ BAP, 0.1 mg L^−1^ IBA, 30 g L^−1^ sucrose, 100 mg L^−1^ myo-inositol (Sigma), and 7.0 g L^−1^ agar were used as the explant source. Before the explants were transferred to the experimental media directly, the plantlets were pre-cultured on hormone-free media for 15–20 days for sterility screening. The plantlets (in the second subculture) were grown on hormone-free media under the same conditions described above, and were transferred to the prepared experimental media.

**Table 1:** **Ion concentrations of the different culture media used for G × N15 rootstock micropropagation in first set of experiments**.

**Ion concentrations (mM)**												
**Media**	$NO_{3}^{-}$	$NH_{4}^{+}$	**K^+^**	**Ca^2+^**	**Mg^2+^**	$SO_{4}^{2-}$	$PO_{4}^{2-}$	**Cl^−^**	**Number**	**Height**	**Callus**	**Quality**
1	37.62	10.31	24.43	1.91	1.13	1.37	0.94	0	10.6 ± 0.4	2.30 ± 0.05	0.07 ± 0.005	4.5 ± 0.13
2	37.62	10.31	25.05	1.91	1.13	1.37	1.56	0	9.8 ± 0.2	2.10 ± 0.04	0.10 ± 0.005	4.2 ± 0.10
3	37.62	10.31	24.43	1.91	1.88	2.12	0.94	0	9.4 ± 0.2	2.02 ± 0.03	0.07 ± 0.003	4.4 ± 0.10
4	37.62	10.31	25.05	1.91	1.88	2.12	1.56	0	9.6 ± 0.4	1.82 ± 0.05	0.12 ± 0.005	4.0 ± 0.07
5	40.15	10.31	24.43	3.18	1.13	1.37	0.94	0	12.6 ± 0.4	2.48 ± 0.03	0.04 ± 0.005	4.9 ± 0.10
6	40.15	10.31	25.05	3.18	1.13	1.37	1.56	0	11.2 ± 0.3	2.32 ± 0.02	0.08 ± 0.005	4.7 ± 0.10
7	40.15	10.31	24.43	3.18	1.88	2.12	0.94	0	9.8 ± 0.3	2.30 ± 0.04	0.06 ± 0.006	4.8 ± 0.12
8	40.15	10.31	25.05	3.18	1.88	2.12	1.56	0	9.2 ± 0.3	2.20 ± 0.05	0.10 ± 0.003	4.7 ± 0.06
9	42.77	15.47	24.43	1.91	1.13	1.37	0.94	0	7.6 ± 0.2	1.82 ± 0.03	0.12 ± 0.003	3.6 ± 0.06
10	42.77	15.47	25.05	1.91	1.13	1.37	1.56	0	6.8 ± 0.2	1.62 ± 0.03	0.15 ± 0.005	3.3 ± 0.11
11	42.77	15.47	24.43	1.91	1.88	2.12	0.94	0	5.8 ± 0.2	1.71 ± 0.03	0.13 ± 0.006	3.2 ± 0.12
12	42.77	15.47	25.05	1.91	1.88	2.12	1.56	0	5.6 ± 0.2	1.47 ± 0.03	0.20 ± 0.008	3.1 ± 0.18
13	45.31	15.47	24.43	3.18	1.13	1.37	0.94	0	8.8 ± 0.2	2.08 ± 0.02	0.11 ± 0.003	3.4 ± 0.16
14	45.31	15.47	25.05	3.18	1.13	1.37	1.56	0	8.2 ± 0.3	1.88 ± 0.02	0.14 ± 0.002	3.4 ± 0.12
15	45.31	15.47	24.43	3.18	1.88	2.12	0.94	0	7.6 ± 0.2	2.34 ± 0.04	0.11 ± 0.002	3.5 ± 0.13
16	45.31	15.47	25.05	3.18	1.88	2.12	1.56	0	7.4 ± 0.2	2.10 ± 0.04	0.15 ± 0.004	3.1 ± 0.06
17	42.31	10.31	29.13	1.91	1.13	1.37	0.94	0	9.4 ± 0.2	2.56 ± 0.02	0.07 ± 0.002	4.4 ± 0.28
18	42.31	10.31	29.75	1.91	1.13	1.37	1.56	0	8.6 ± 0.2	2.36 ± 0.01	0.11 ± 0.004	3.7 ± 0.18
19	42.31	10.31	29.13	1.91	1.88	2.12	0.94	0	8.4 ± 0.2	2.42 ± 0.03	0.09 ± 0.002	4.0 ± 0.08
20	42.31	10.31	29.75	1.91	1.88	2.12	1.56	0	7.6 ± 0.2	2.28 ± 0.02	0.12 ± 0.004	3.6 ± 0.05
21	44.85	10.31	29.13	3.18	1.13	1.37	0.94	0	11 ± 0.3	2.54 ± 0.01	0.06 ± 0.002	4.4 ± 0.23
22	44.85	10.31	29.75	3.18	1.13	1.37	1.56	0	9.6 ± 0.4	2.64 ± 0.02	0.10 ± 0.006	4.1 ± 0.13
23	44.85	10.31	29.13	3.18	1.88	2.12	0.94	0	9.6 ± 0.2	2.44 ± 0.02	0.08 ± 0.003	4.4 ± 0.13
24	44.85	10.31	29.75	3.18	1.88	2.12	1.56	0	9 ± 0.3	2.64 ± 0.02	0.11 ± 0.006	3.6 ± 0.06
25	47.74	15.47	29.13	1.91	1.13	1.37	0.94	0	7.6 ± 0.2	2.28 ± 0.02	0.13 ± 0.006	3.5 ± 0.11
26	47.74	15.47	29.75	1.91	1.13	1.37	1.56	0	8 ± 0.3	2.06 ± 0.05	0.17 ± 0.006	3.2 ± 0.12
27	47.74	15.47	29.13	1.91	1.88	2.12	0.94	0	7 ± 0.3	1.86 ± 0.04	0.15 ± 0.006	3.5 ± 0.09
28	47.74	15.47	29.75	1.91	1.88	2.12	1.56	0	6 ± 0.3	1.64 ± 0.02	0.19 ± 0.007	3.3 ± 0.11
29	50.01	15.47	29.13	3.18	1.13	1.37	0.94	0	9.6 ± 0.4	2.06 ± 0.02	0.09 ± 0.006	4.1 ± 0.10
30	50.01	15.47	29.75	3.18	1.13	1.37	1.56	0	9.2 ± 0.3	2.28 ± 0.02	0.12 ± 0.004	3.7 ± 0.10
31	50.01	15.47	29.13	3.18	1.88	2.12	0.94	0	8.6 ± 0.2	2.36 ± 0.02	0.10 ± 0.005	4.1 ± 0.13
32	50.01	15.47	29.75	3.18	1.88	2.12	1.56	0	7.6 ± 0.2	2.57 ± 0.03	0.13 ± 0.009	3.5 ± 0.11
33	33.8	10.31	24.74	2.99	1.5	1.74	1.25	2.99	10.2 ± 0.3	2.02 ± 0.05	0.10 ± 0.005	4.0 ± 0.22
34	38.95	15.47	24.74	2.99	1.5	1.74	1.25	2.99	7.2 ± 0.3	1.62 ± 0.03	0.23 ± 0.009	3.3 ± 0.11
35	38.5	10.31	29.44	2.99	1.5	1.74	1.25	2.99	8.6 ± 0.2	2.20 ± 0.07	0.13 ± 0.005	3.7 ± 0.15
36	43.65	15.47	29.44	2.99	1.5	1.74	1.25	2.99	7.8 ± 0.3	1.86 ± 0.04	0.19 ± 0.009	3.3 ± 0.11
MS	39.41	20.62	20.04	3.96	1.5	1.74	1.25	2.99	8.8 ± 0.4	2.4 ± 0.04	0.16 ± 0.005	3.4 ± 0.06
EM	49.62	31.28	12.41	3.39	0.41	0.65	0.85	0	6.8 ± 0.2	1.66 ± 0.05	0.20 ± 0.009	3.0 ± 0.09
WPM	9.71	5.0	6.93	3.0	1.5	7.42	1.25	0.65	6.4 ± 0.2	1.51 ± 0.02	0.17 ± 0.004	4.3 ± 0.11

**Table 2:** **Ion concentrations of the different culture media used for G × N15 rootstock micropropagation in second set of experiments**.

**Ion concentrations (mM)**												
**Media**	$NO_{3}^{-}$	$NH_{4}^{+}$	**K^+^**	**Ca^2+^**	**Mg^2+^**	$SO_{4}^{2-}$	$PO_{4}^{2-}$	**Cl^−^**	**Number**	**Height**	**Callus**	**Quality**
1	17.93	10.31	8.04	3.81	1.13	8.47	0.94	0	5.2 ± 0.32	1.34 ± 0.05	0.09 ± 0.005	3.7 ± 0.10
2	17.93	10.31	8.66	3.81	1.13	8.47	1.56	0	5.0 ± 0.32	1.49 ± 0.03	0.10 ± 0.005	3.6 ± 0.06
3	17.93	10.31	8.04	3.81	1.88	9.22	0.94	0	6.2 ± 0.38	1.59 ± 0.04	0.10 ± 0.008	4.2 ± 0.09
4	17.93	10.31	8.66	3.81	1.88	9.22	1.56	0	6.8 ± 0.20	1.70 ± 0.07	0.13 ± 0.005	4.0 ± 0.12
5	23.01	10.31	8.04	6.35	1.13	8.47	0.94	0	7.4 ± 0.40	1.81 ± 0.03	0.07 ± 0.009	4.4 ± 0.10
6	23.01	10.31	8.66	6.35	1.13	8.47	1.56	0	8.0 ± 0.32	1.96 ± 0.05	0.10 ± 0.008	4.2 ± 0.06
7	23.01	10.31	8.04	6.35	1.88	9.22	0.94	0	8.6 ± 0.25	1.95 ± 0.03	0.08 ± 0.009	4.4 ± 0.10
8	23.01	10.31	8.66	6.35	1.88	9.22	1.56	0	9.2 ± 0.20	1.97 ± 0.05	0.10 ± 0.007	4.2 ± 0.09
9	23.09	15.47	8.04	3.81	1.13	8.47	0.94	0	3.2 ± 0.32	1.12 ± 0.06	0.19 ± 0.011	3.2 ± 0.13
10	23.09	15.47	8.66	3.81	1.13	8.47	1.56	0	3.0 ± 0.32	1.02 ± 0.07	0.25 ± 0.007	3.0 ± 0.08
11	23.09	15.47	8.04	3.81	1.88	9.22	0.94	0	3.4 ± 0.32	1.16 ± 0.05	0.22 ± 0.008	3.1 ± 0.17
12	23.09	15.47	8.66	3.81	1.88	9.22	1.56	0	3.8 ± 0.37	1.10 ± 0.08	0.25 ± 0.009	3.0 ± 0.14
13	28.17	15.47	8.04	6.35	1.13	8.47	0.94	0	4.6 ± 0.24	1.41 ± 0.06	0.12 ± 0.006	3.7 ± 0.09
14	28.17	15.47	8.66	6.35	1.13	8.47	1.56	0	4.8 ± 0.20	1.30 ± 0.07	0.17 ± 0.007	3.5 ± 0.09
15	28.17	15.47	8.04	6.35	1.88	9.22	0.94	0	5.2 ± 0.20	1.53 ± 0.05	0.15 ± 0.011	3.8 ± 0.12
16	28.17	15.47	8.66	6.35	1.88	9.22	1.56	0	5.8 ± 0.37	1.38 ± 0.09	0.19 ± 0.011	3.5 ± 0.09
17	17.93	10.31	9.46	3.81	1.13	9.89	0.94	0	6.4 ± 0.24	1.48 ± 0.09	0.12 ± 0.007	4.6 ± 0.17
18	17.93	10.31	10.08	3.81	1.13	9.89	1.56	0	5.8 ± 0.20	1.58 ± 0.06	0.14 ± 0.009	4.5 ± 0.08
19	17.93	10.31	9.46	3.81	1.88	10.64	0.94	0	7.0 ± 0.00	1.60 ± 0.07	0.13 ± 0.007	4.3 ± 0.09
20	17.93	10.31	10.08	3.81	1.88	10.64	1.56	0	6.8 ± 0.20	1.82 ± 0.06	0.16 ± 0.007	4.3 ± 0.17
21	23.01	10.31	9.46	6.35	1.13	9.89	0.94	0	8.8 ± 2.20	1.94 ± 0.04	0.07 ± 0.012	5.0 ± 0.05
22	23.01	10.31	10.08	6.35	1.13	9.89	1.56	0	8.6 ± 0.40	1.96 ± 0.05	0.11 ± 0.007	4.9 ± 0.06
23	23.01	10.31	9.46	6.35	1.88	10.64	0.94	0	10 ± 0.32	2.10 ± 0.07	0.10 ± 0.009	4.7 ± 0.09
24	23.01	10.31	10.08	6.35	1.88	10.64	1.56	0	9.0 ± 0.45	2.40 ± 0.07	0.13 ± 0.007	4.5 ± 0.09
25	23.09	15.47	9.46	3.81	1.13	9.89	0.94	0	4.2 ± 0.37	1.04 ± 0.08	0.15 ± 0.009	3.5 ± 0.08
26	23.09	15.47	10.08	3.81	1.13	9.89	1.56	0	4.8 ± 0.37	1.06 ± 0.09	0.19 ± 0.007	3.3 ± 0.09
27	23.09	15.47	9.46	3.81	1.88	10.64	0.94	0	5.0 ± 0.32	1.08 ± 0.04	0.16 ± 0.005	3.6 ± 0.09
28	23.09	15.47	10.08	3.81	1.88	10.64	1.56	0	5.8 ± 0.20	1.28 ± 0.07	0.20 ± 0.009	3.2 ± 0.09
29	28.17	15.47	9.46	6.35	1.13	9.89	0.94	0	6.8 ± 0.20	1.22 ± 0.07	0.13 ± 0.009	3.9 ± 0.13
30	28.17	15.47	10.08	6.35	1.13	9.89	1.56	0	6.4 ± 0.25	1.55 ± 0.06	0.15 ± 0.011	3.8 ± 0.08
31	28.17	15.47	9.46	6.35	1.88	10.64	0.94	0	7.6 ± 0.25	1.59 ± 0.07	0.15 ± 0.008	3.7 ± 0.09
32	28.17	15.47	10.08	6.35	1.88	10.64	1.56	0	7.0 ± 0.32	1.67 ± 0.05	0.17 ± 0.009	3.5 ± 0.09
33	10.31	10.31	8.35	2.99	1.50	8.84	1.25	3.0	7.8 ± 0.58	2.07 ± 0.04	0.11 ± 0.007	3.9 ± 0.13
34	15.47	15.47	8.35	2.99	1.50	8.84	1.25	3.0	7.2 ± 0.66	1.43 ± 0.04	0.21 ± 0.011	3.1 ± 0.13
35	10.31	10.31	9.77	2.99	1.50	10.26	1.25	3.0	9.4 ± 0.68	2.26 ± 0.08	0.16 ± 0.007	4.5 ± 0.08
36	15.47	15.47	9.77	2.99	1.50	10.26	1.25	3.0	7.0 ± 0.32	1.34 ± 0.16	0.18 ± 0.005	3.4 ± 0.06

Before starting the present investigation several experiments have been done for choosing the best media culture among common media cultures (unpublished). In the first stage of the study four media culture commonly used for *prunus* rootstock micropropagation were investigated (unpublished data). In the second stage the best medium cultures (MS and WPM basal medium) were selected for prediction and optimization of specific GN15 micropropagation media culture. Afterwards the critical elements in selected media have been investigated and determined the vital and essential elements for proliferation stage. In the present investigation we have just investigated the most important elements that have been selected from previous steps. finally two single factorial experiments were designed to discover the best macro element combinations for GN rootstock proliferation. In the next stage data from both experiments were analyzed simultaneously by ANN-GA. Details of the tests and modifications on the basic culture media are fully described below.

### First set of experiments

To optimize a new culture medium for proliferation, 36 media were designed to evaluate the effects of the main macronutrients on G × N5 hybrid rootstock micropropagation. Five mineral nutrient factors used were based on MS salts, with each factor varied over a range of concentrations (× MS): 1.25 × and 1.5 × for KNO_3_ (2375 and 2850 mg L^−1^); 0. 5 × and 0.75 × for NH_4_NO_3_ (825 and 1238 mg L^−1^); 0.75 × and 1.25 × for Ca(NO_3_)_2_·4H_2_O (450 and 750 mg L^−1^); 0.75 × and 1.25 × for MgSO_4_·7H_2_O (278 and 463 mg L^−1^) and 0.75 × and 1.25 × for KH_2_PO_4_ (128 and 213 mg L^−1^). They were tested in multifactorial combinations, and also four MS-modified media containing CaCl_2_ were used (Table 1). The micronutrient and vitamin concentrations were the same for all designed culture media, and were the same as for those described for MS medium (Murashige and Skoog, 1962). As controls, we used other media, such as MS (Murashige and Skoog, 1962), EM and WPM (Lloyd and McCown, 1981) media that have also been generally used in *Prunus* sp.

### Second set of experiments

In order to optimize a new culture medium, 36 different media were used to evaluate the effect of macronutrients on proliferation of G × N15 hybrid rootstock. The five mineral nutrient factors used in these experiments were based on MS and WPM salts, with each factor varied over a range of concentrations (× MS and × WPM): 1.25 × and 1.5 × for K_2_SO_4_ (1237 and 1485 mg L^−1^); 0.5 × and 0.75 × for NH_4_NO_3_ (825 and 1238 mg L^−1^); 0.75 × and 1.25 × for Ca(NO_3_)_2_·4H_2_O (450 and 750 mg L^−1^); 0.75 × and 1.25 × for MgSO_4_·7H_2_O (278 and 463 mg L^−1^) and 0.75 × and 1.25 × for KH_2_PO_4_ (128 and 213 mg L^−1^). These factors were tested in multifactorial combinations (Table 2). The micronutrient and vitamin concentrations were the same for all designed culture media, and were the same as for those described for MS medium (Murashige and Skoog, 1962). As controls, we used four modified WPM and MS media containing different concentrations of NH_4_NO_3_, K_2_SO_4_, and CaCl_2_ to facilitate comparisons (Table 2).

### Third set of experiments

In this experiment, five culture media were employed: MS medium (Murashige and Skoog, 1962), WPM (Lloyd and McCown, 1981), QL (Quoirin and Lepoivre, 1977), EM, and modified MS medium (YAS) containing the predicted and optimized mineral nutrients based on ANN-GA. The media were supplemented with 1 mg L^−1^ BAP, 0.1 mg L^−1^ IBA, 30 g L^−1^ sucrose and 100 mg L^−1^ myo-inositol (Sigma), and the *pH*-values of all media were adjusted to 5.7–5.8 prior to the addition of the gelling agent (7.0 g L^−1^ agar).

### Culture environment

The cultures were subjected to a 16/8-h (light/dark) photoperiod at a light intensity of 80 μmol m^−2^s^−1^ provided by white fluorescent tubes in a growth chamber (25°C), and sub-cultured for 30 days.

### Experimental design and data collection

The first two experiments were conducted using a factorial based on a completely randomized design (CRD) with five replications; each replication included four explants in one glass baby food jar per treatment. The third experiment, which was also performed using a CRD, was repeated and the reported data are the means of the results of the three trials. The cultures were continuously observed for any response, and data were collected after 4 weeks. At the end of the *in vitro* proliferation stage, four parameters (outputs) were recorded to analyze the effects of the variables (inputs) on proliferation: (1) total shoots produced (number of new microshoots per explant); (2) lengths of microshoots (cm), but only for new shoots longer than 0.75 cm; (3) callus weight, with the callus derived from the base of stem explants; and (4) a subjective rating of the quality of the plant appearance scored 1–5 based on growth parameters such as vitrification, shoot tip necrosis, yellowing, leaf area and leaf quality (1 = poor quality, 2 = acceptable, 3 = medium, 4 = good, and 5 = very good or best quality; Niedz and Evens, 2007). To derive a formulation of a new culture medium for G × N15 *in vitro* proliferation, the first two experiments were combined and analyzed using the artificial neural network genetic algorithm (ANN-GA) introduced above. Commercially available software, Matlab® R2010a (Matlab, 2010), was used to write the mathematical code for developing and evaluating the ANN model. The developed program is actually a modification of the source code of an ANN algorithm that was previously applied by Ahmadi and Golian (2011). In the third experiment to assess the efficiency of ANN-GA in predicting and optimizing the new formulated medium (YAS), comparisons of this new medium (YAS) with MS, WPM, EM, and QL basal salts were carried out. Data from this third experiment were subjected to a one-way analysis of variance (ANOVA). Significance was determined by analysis of the variance, and significant (i.e., *P* ≤ 0.05) differences between mean values were estimated using the least significant difference test. SAS version 9.1 was used for statistical analyses.

### ANN-GA model

In the present study, one of the most well-known network algorithms, the feed forward back-propagation learning algorithm, including input, output and hidden layers (a three-layer back-propagation network) was applied in developing the ANN model (Demuth et al., 2006; Ahmadi and Golian, 2011). The transfer function included hyperbolic tangent sigmoid (tansig) and linear (purelin) functions for the hidden and output layers, respectively. For training the network, a Levenberg-Marquardt algorithm was used for back-propagation with a gradient descent with momentum weight and bias learning function (Demuth et al., 2006). The 0.01 level MS error was used as the performance function, and training was terminated after 1000 epochs or iterations of the network. In ANN process, eight inputs and the four outputs were employed. Eight inputs variables corresponding to different levels of ${\text{NH}}_{4}^{+}$, ${\text{N0}}_{3}^{-}$, K^+^, Ca^2+^, Mg^2+^, ${\text{SO}}_{4}^{2-}$, ${\text{PO}}_{4}^{2-}$, and Cl^−^ were used as units in the ANN model input layer. Four models were developed separately for the number of microshoots, lengths of the microshoots, callus rate, and quality index. The 375 data sets generated from the first two factorials based on CRDs were used to train and test the network. Before training, each data set (input and output data) was normalized (−1, 1) in order to make the problem simpler for the network, to obtain fast convergence minimum mean square error, and to ensure that the targets (output data) would reproducibly fall into the specific range of the new feed forward network (Demuth et al., 2006; Gulati et al., 2010). For artificial neural network modeling, 225 (i.e., c. 60%) of the data samples were randomly selected for use as training samples, with another 150 data sets used as testing samples. Training data were used to present the cause–effect relationship for the model to learn, and test data were for assessing the quality of the model.

**Figure 1:**
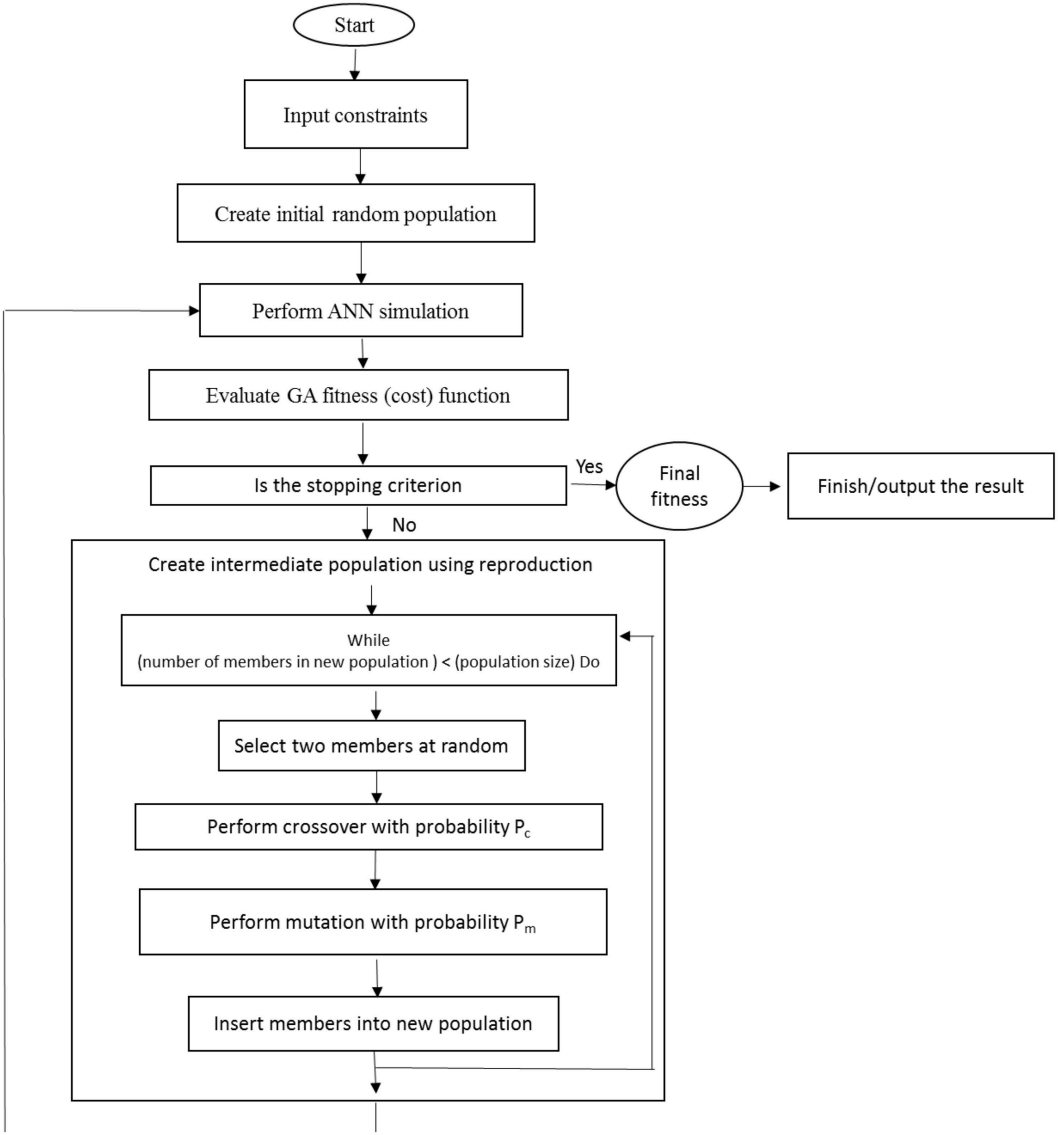
**Schematic describing the relationship between ANN and GA**.

After the training process, the developed ANN models were subjected to additional practice using GA to find the optimal values of the input variables (the ${\text{NH}}_{4}^{+}$, ${\text{NO}}_{3}^{-}$, K^+^, Ca^2+^, Mg^2+^, ${\text{SO}}_{4}^{2-}$, ${\text{PO}}_{4}^{2-}$, and Cl^−^ requirements) to maximize the number of microshoots, length of microshoots and quality index, as well as to minimize callus rate. The input vector comprising of input variables of model becomes the decision variable for the GA. The GA treats an optimization using a cycle of four stages of initialization of solution populations, fitness computation based on objective function, selection of best chromosomes, and genetic propagation of selected parent chromosomes using genetic operators like crossover and mutation to create the new population of chromosomes (Desai et al., 2008). The whole process continues until a suitable result is achieved (Figure 1). The chromosome is a collection of genes where genes were represented by binary encoding method. A “*roulette wheel selection*” method was used for selecting elite populations for crossover. Initial population of 50, generation number of 500, mutation rate of 0.1, and crossover rate of 0.85 has been set to obtain the best fitness. This generational process was repeated until the number of generations has been reached. During GA implementation, the search for the optimal solutions was restricted between the input variable bounds specified in CRD design (Tables 1, 2).

Sensitivity analysis was performed on the developed ANN models to determine which input variable would be more effective in the model output. In other words, such sensitivity analyses of the models would determine which of the ${\text{NH}}_{4}^{+}$, ${\text{NO}}_{3}^{-}$, K^+^, Ca^2+^, Mg^2+^, ${\text{SO}}_{4}^{2-}$, ${\text{PO}}_{4}^{2-}$, and Cl^−^ ions tested is most effective to achieve optimal number of microshoots, length of microshoots, callus rate and quality index in proliferation of G × N15. The sensitivities of output vs. each input variables were assessed by determining the variable sensitivity error (VSE) value, which shows the performance of the developed ANN model if that variable is unavailable, and the value of the variable sensitivity ratio (VSR), which is a relative indication of the ratio between the VSE and ANN model error when all variables are available (Lou and Nakai, 2001; Ahmadi and Golian, 2010a,b). A more important variable has a higher VSR value. Thus, based on the obtained VSR value, the input variables may be ranked in order of importance.

Actual and predicted output values were compared using criteria that are commonly used to evaluate forecasting models. The accuracy of each model was assessed using the root mean squared error (RMSE) and the *R*^2^ for each output. The *R*^2^ values which was used to investigate the efficiency of each model, was computed using the equation

$$R^{2}\;=\;1-\frac{\sum_{\text{i }=\;1}^{\text{n}}{\left({\text{y}}_{\text{i}}-{\hat{\text{y}}}_{i}\right)}^{2}}{\sum_{\text{i }=\;1}^{\text{n}}{\left({\text{y}}_{\text{i}}-{\bar{\text{y}}}_{i}\right)}^{2}}$$

The RMSE was computed using the equation

$$\text{RMSE }=\;\sqrt{\;\frac{\sum_{\text{i }=\;1}^{\text{n}}\;{\left|\hat{\text{y}}-\;{\text{y}}_{\text{i}}\right|}^{2}}{\text{n}}}$$

where n is number of observations, y_i_ is the observed value of dependent variable, ${\hat{\text{y}}}_{\text{i}}$ is the predicted value from the model, and y_i_ is average observed values of each output. The *R*^2^ ratio describes how much of the variance of the parameters (dependent variable) is accounted for in the model: The larger the value of the training set *R*^2^, the more the model captured the variation in the training data. The values predicted by the ANN models are plotted against the corresponding experimental values to evaluate the modeling abilities.

Matlab R2010a (Matlab, 2010) software was used to write mathematical code to develop and evaluate the ANN-GA model.

## Results

### Validation experiment: assessment of the optimum productivity produced by the new medium formulation (YAS)

Multifactorial analysis of variance showed that the MS, EM, QL, WPM, and YAS media had significantly different effects (*P* < 0.001) on the proliferation rate, shoot length, callus weight derived from the base of stem and quality index of GN-15 rootstock after 4 weeks in culture (Table 3). The best shoot formation was obtained on MS medium. This medium, when including 1 mg l^−1^ BAP and 0.1 mg l^−1^ IBA, resulted in the production of an average of 10.25 microshoots per explant inoculated, which is significantly higher than that produced by using the same concentrations of BAP and IBA in other media (Table 3). MS and YAS were the most productive media, and the QL and WPM media were the poorest performers for this rootstock. The maximum average shoot length (1.99 cm) obtained using YAS medium was also significantly greater than that using MS, EM, QL, and WPM media (Table 3). WPM medium supplemented with 1 mg l^−1^ BAP and 0.1 mg l^−1^ IBA resulted in production of the highest callus weight derived from the base of the stem explants (0.20 g per explant), which was significantly higher than that produced by using other media (Table 3). The result also showed that the concentration of ${\text{NH}}_{4}^{+}$ correlated inversely to the quality index. On the other hand, increasing the ${\text{NH}}_{4}^{+}$ concentration led to a decrease in the quality index. The best plantlet quality index, 4.69, was obtained using the WPM medium, and this value was also significantly better than the indexes obtained using MS and EM media (Table 3). The MS medium resulted in the production of an average of 10.25 microshoots per explant inoculated, which is significantly higher than that produced by the use of other media, but the highest average micro-shoot length (1.84 cm) and plantlet quality index (4.75) from the MS medium were significantly lower than those from the YAS medium (Table 3). Based on the above results, the MS medium produced an undesirable plantlet quality and mean microshoot length, and this medium was less efficient than the YAS medium, but it did produce the most microshoots. It can be concluded that YAS medium was overall superior to MS, EM, QL, and WPM media for proliferation of the G × N15 rootstock.

**Table 3:** **Effect of different culture media on Shoot Number, Shoot length, callus rate, and quality index**.

**Effects**	**Proliferation**	**Length**	**Callus**	**Quality index**
**MEDIUM**				
MS	10.25 ± 0.25 a	1.84 ± 0.01 b	0.16 ± 0.003 b	3.34 ± 0.10 b
EM	8.63 ± 0.26 b	1.55 ± 0.01 c	0.19 ± 0.005 a	3.25 ± 0.12 b
QL	7.25 ± 0.25 c	1.60 ± 0.01 c	0.13 ± 0.004 c	4.34 ± 0.11 a
WPM	6.00 ± 0.27 d	1.39 ± 0.01 d	0.20 ± 0.003 a	4.69 ± 0.06 a
YAS	9.25 ± 0.25 ab	1.99 ± 0.01 a	0.06 ± 0.005 d	4.31 ± 0.06 a
***P*****-VALUE**				
Medium	< 0.001	< 0.001	< 0.001	< 0.001

*Values in each column represent means ±SE. Different letters within columns indicate significant differences (p < 0.05)*.

### ANN-GA modeling and evaluation

While many studies have concentrated on the micropropagation of *Prunus* rootstock, the formulation of effective media for such rootstock has received much less attention. However, optimization or modification of media based on appropriately selecting minerals dramatically influences the development and growth of shoot, including the shoot growth (length), proliferation (number of new microshoots), weight of calluses derived from the base of stem explants and quality index. In the present study, the response of GN-15 rootstocks was found to depend on how the mineral nutrients were modified in the MS medium. MS, WPM, and QL media were usually used as the standard growth media for all *Prunus* rootstocks and, whenever studied, the formulations of these media were predicted and optimized with the concentrations of these mineral ions taken into consideration as factors. An extremely easy-to-use commercial neural network software, Matlab R2010a (Matlab, 2010), and the genetic algorithm, ANN-GA, succeeded in simultaneously modeling and optimizing the four growth parameters mentioned above that is, the number of microshoots, length of microshoots, callus weight, and quality index selected as a function of the concentrations of eight ions (inputs).

**Figure 2:**
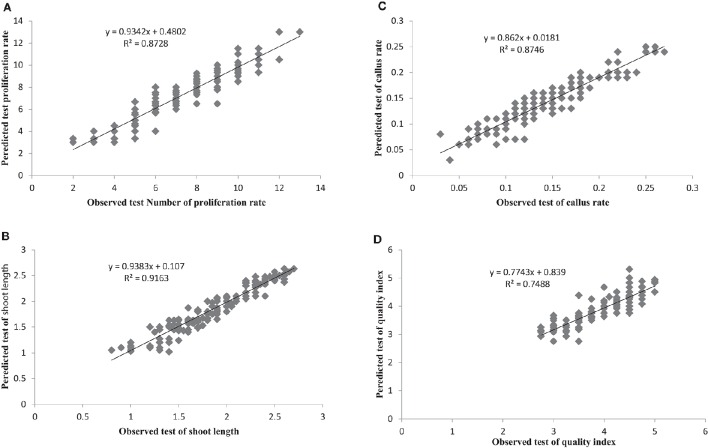
**Scatter plot of observed vs. model-predicted values of (A) Proliferation rate, (B) shoot length, (C) Callus rate, and (D) quality index of G × N15 rootstock during ***in vitro*** multiplication obtained by artificial neural network model testing set (***n*** = 150)**. The solid line indicates the fitted simple regression line on scatter points.

The most important objective of ANN-based modeling approaches is to develop a model that as precisely as possible predicts value(s) of the output variable(s). Comparing the observed and predicted output values may describe the performance of the ANN model based on the investigated inputs. The predicted ANN model graph vs. observed values for the number of microshoots, length of microshoots, callus weight and quality index are shown in Figure 2. The fitted simple regression lines indicate good agreement between the observed and predicted values for all four of these growth parameters, for both the training and testing sets. Using a high squared correlation coefficient fitting method and based on the ANN models derived, four graphs were produced to display how each of the four growth parameters varied as the percentages of ${\text{NH}}_{4}^{+}$, ${\text{NO}}_{3}^{-}$, ${\text{PO}}_{4}^{2-}$, Ca^2+^, K^+^, ${\text{SO}}_{4}^{2-}$, Mg^2+^, and Cl^−^ were varied (Figure 2).

The graphs may be useful for understanding the complete relationship between nutrients and responses, and to evaluate the combined effects of modifying the mineral nutrients in the MS medium. The goodness-of-fit statistical values derived from the ANN model to predict the number of microshoots, length of microshoots, callus weight and quality index are shown in **Table 6**. The ANN models were able to accurately (*R*^2^ > 87, 91, 87, and 74) predict each of these four growth parameters in the testing data sets, which were not used during the training processes (Figure 2). Moreover, the trained ANN models of these four growth parameters yielded balanced statistics for both the training and testing subsets (**Table 6**). Overall, the statistics (**Table 6**) revealed that the ANN-based models could efficiently fit published data on the responses of G × N15 microshoots during *in vitro* multiplication to the MS medium with modified mineral nutrients.

### Sensitivity analysis of the models

The relative importance of input variables was determined using the entire 375 lines of data (training and testing) to calculate the overall VSR. The VSR values obtained for the model output, i.e., number of microshoots, length of microshoots, callus weight and quality index, with respect to changes in the mineral nutrients of the MS medium are shown in Table 4. The number of microshoots of G × N15 was found to be most sensitive to the concentration of ${\text{NH}}_{4}^{+}$ (VSR = 6.7), followed by ${\text{NO}}_{3}^{-}$ (VSR = 6.3), Ca^2+^ (VSR = 4.2), ${\text{PO}}_{4}^{2-}$ (VSR = 4), K^+^ (VSR = 2.6), Mg^2+^ (VSR = 2.2), ${\text{SO}}_{4}^{2-}$ (VSR = 2.2), and Cl^−^ (VSR = 2.1) (Table 4). For the length of microshoots model, the feed efficiency of G × N15 also showed the most sensitivity to ${\text{NH}}_{4}^{+}$ concentration (VSR = 9.8), followed by ${\text{PO}}_{4}^{2-}$ (VSR = 7.7), ${\text{NO}}_{3}^{-}$ (VSR = 6.9), Ca^2+^ (VSR = 6.6), K^+^ (VSR = 3.4), Mg^2+^ (VSR = 2.6), ${\text{SO}}_{4}^{2-}$ (VSR = 2.5), and Cl^−^ (VSR = 2.1) (Table 4). In micropropagation, the aim is to reduce callus since it results in somaconal variation. The callus weight of G × N15 was also found to be most sensitive to the concentration of ${\text{NH}}_{4}^{+}$ (VSR = 11.4), followed by ${\text{NO}}_{3}^{-}$ (VSR = 10.4), ${\text{PO}}_{4}^{2-}$ (VSR = 10.1), Ca^2+^ (VSR = 3.7), K^+^ (VSR = 2.8), ${\text{SO}}_{4}^{2-}$ (VSR = 2.3), Cl^−^ (VSR = 2.2), and Mg^2+^ (VSR = 1.8) (Table 4). For the quality index model, the feed efficiency of G × N15 plantlets showed most sensitivity to the concentration of ${\text{NO}}_{3}^{-}$ (VSR = 5.8), followed by ${\text{NH}}_{4}^{+}$ (VSR = 5.5), K^+^ (VSR = 2.3), ${\text{PO}}_{4}^{2-}$ (VSR = 2.3), Cl^−^ (VSR = 2.2), ${\text{SO}}_{4}^{2-}$ (VSR = 1.9), Ca^2+^ (VSR = 1.8), and Mg^2+^ (VSR = 1.4) (Table 4). These results suggests that ion concentrations (inputs) can significantly influence the performance of G × N15 multiplication; however, the effects of ${\text{NH}}_{4}^{+}$, ${\text{NO}}_{3}^{-}$, ${\text{PO}}_{4}^{2-}$, Ca^2+^, and K^+^ levels were more pronounced than were the effects of ${\text{SO}}_{4}^{2-}$, Mg^2+^, and Cl^−^ levels. Several researchers have suggested that the responses of *Prunus* rootstock to ${\text{NH}}_{4}^{+}$, ${\text{NO}}_{3}^{-}$, ${\text{PO}}_{4}^{2-}$, Ca^2+^, K^+^, ${\text{SO}}_{4}^{2-}$, Mg^2+^, and Cl^−^ differ from one another. Significant effects of ${\text{NH}}_{4}^{+}$, ${\text{NO}}_{3}^{-}$, ${\text{PO}}_{4}^{2-}$, Ca^2+^, and K^+^ on *Prunus* rootstock multiplication performance have been reported by previous researchers, but the effects of the concentrations of these ions on the responses of plants usually depend on the experimental design and how the statistical evaluation was applied. On the other hand, appropriate mathematical or statistical models are necessary to extract appropriate conclusions regarding the response to ion levels. Individual experiments may show no significant response to ions levels. However, when the data from several experiments are pooled together and analyzed with an appropriate model, the responses to ion concentration may be highly significant.

**Table 4:** **Importance of ion concentrations (mM) of the different culture media used for G × N15 rootstock micropropagation according to the sensitivity analysis on the developed neural network model to rank the importance of ion concentrations**.

**VSR**								
**Element**	$NO_{3}^{-}$	$NH_{4}^{+}$	**K^+^**	**Ca^2+^**	**Mg^2+^**	$SO_{4}^{2-}$	$PO_{4}^{2-}$	**Cl^−^**
Proliferation rate	6.3	6.7	2.6	4.2	2.3	2.2	4	2.1
Rank	2	1	5	3	6	7	4	8
Shoot length	6.9	9.8	3.4	6.6	2.6	2.5	7.7	2.1
Rank	3	1	5	4	6	7	2	8
Callus rate	10.4	11.4	2.8	3.7	1.8	2.3	10.1	2.2
Rank	2	1	5	4	8	6	3	7
Quality index	5.8	5.5	2.3	1.8	1.4	1.9	2.3	2.2
Rank	1	2	3	6	7	5	3	4

*VSR, relative indication of the ratio between the variable sensitivity error and the error of the model when all variables are available*.

### Model optimization

The final aim of the current study was to analyze the ANN models to address the question of what levels of ${\text{NH}}_{4}^{+}$, ${\text{NO}}_{3}^{-}$, K^+^, Ca^2+^, Mg^2+^, ${\text{SO}}_{4}^{2-}$, ${\text{PO}}_{4}^{2-}$, and Cl^−^ should be used to achieve the maximum number and length of microshoots and maximum quality index, as well as the minimum callus weight of G × N15 rootstock. Our aim not only was to predict a new culture medium but also was to optimize and use this new medium. The results of our optimization are summarized in Table 5. The optimization process was conducted in the range of values found in the data sets (Tables 1, 2). The optimization analysis on the ANN model to maximize the number of microshoots of G × N15 rootstock under the *in vitro* multiplication condition revealed that the maximum number of microshoots may be obtained with a medium containing 27.5 mM of ${\text{NO}}_{3}^{-}$, 14 mM of ${\text{NH}}_{4}^{+}$, 25.9 mM of K^+^, 5 mM of Ca^2+^, 0.7 mM of Mg^2+^, 4.7 mM of ${\text{SO}}_{4}^{2-}$, 1.1 mM of ${\text{PO}}_{4}^{2-}$, and 0.96 mM of Cl^−^ (Table 5). The number of microshoots at this optimal point was predicted to be 12. The longest microshoots may be achieved with a medium supplemented with 48.9 mM of ${\text{NO}}_{3}^{-}$, 6.9 mM of ${\text{NH}}_{4}^{+}$, 28.8 mM of K^+^, 4.9 mM of Ca^2+^, 1.5 mM of Mg^2+^, 4.3 mM of ${\text{SO}}_{4}^{2-}$, 1.3 mM of ${\text{PO}}_{4}^{2-}$, and 0 mM of Cl^−^, for which the microshoots length was predicted to be 2.63 cm (Table 5). The optimization analysis on the ANN model to minimize the callus weight of G × N15 rootstock under the *in vitro* multiplication condition revealed that the minimum callus weight may be obtained with a medium containing 28.2 mM of ${\text{NO}}_{3}^{-}$, 17.6 mM of ${\text{NH}}_{4}^{+}$, 16.3 mM of K^+^, 4.5 mM of Ca^2+^, 1.4 mM of Mg^2+^, 2.6 mM of ${\text{SO}}_{4}^{2-}$, 1.2 mM of ${\text{PO}}_{4}^{2-}$, and 0.07 mM of Cl^−^ (Table 5). The callus weight was predicted to be 0.03 (gr) at this optimal condition. The optimal plantlet quality may be achieved with the medium being supplemented with 13.3 mM of ${\text{NO}}_{3}^{-}$, 5.8 mM of ${\text{NH}}_{4}^{+}$, 28.2 mM of K^+^, 4.5 mM of Ca^2+^, 0.89 mM of Mg^2+^, 7.6 mM of ${\text{SO}}_{4}^{2-}$, 0.9 mM of ${\text{PO}}_{4}^{2-}$, and 1 mM of Cl^−^ (Table 5), for which the plantlet quality index was predicted to be 4.95. The ion concentrations suggested by the ANN model showed that the required ${\text{NO}}_{3}^{-}$ and ${\text{NH}}_{4}^{+}$ concentrations for optimal productivity in *in vitro* multiplication of G × N15 rootstock were lower than those concentrations on MS medium. In conclusion, a platform of ANN-based models with sensitivity analysis and optimization algorithms was used successfully in this study to integrate published data on the responses of *in vitro* multiplication of G × N15 rootstock to macro element nutrient concentration. Analyses of the ANN models for the number of microshoots and the lengths of microshoots from a compiled data set suggested that the concentrations of ${\text{NH}}_{4}^{+}$, ${\text{NO}}_{3}^{-}$, ${\text{PO}}_{4}^{2-}$, Ca^2+^, and K^+^ were more important than the concentrations of ${\text{SO}}_{4}^{2-}$, Mg^2+^, and Cl^−^. The results revealed that a medium containing 27.5 mM of ${\text{NO}}_{3}^{-}$, 14 mM of ${\text{NH}}_{4}^{+}$, 25.9 mM of K^+^, 5 mM of Ca^2+^, 0.7 mM of Mg^2+^, 4.7 mM of ${\text{SO}}_{4}^{2-}$, 1.1 mM of ${\text{PO}}_{4}^{2-}$, and 0.96 mM of Cl^−^ (Table 5) may lead to the production of optimal microshoots, whereas the optimal length of microshoots may be achieved with a medium containing 48.9 mM of ${\text{NO}}_{3}^{-}$, 6.9 mM of ${\text{NH}}_{4}^{+}$, 28.8 mM of K^+^, 4.9 mM of Ca^2+^, 1.5 mM of Mg^2+^, 4.3 mM of ${\text{SO}}_{4}^{2-}$, 1.3 mM of ${\text{PO}}_{4}^{2-}$, and 0 mM of Cl^−^ (Table 5). According to the preliminary results obtained by the ANN-GA, optimal productivity (number of new microshoots × length of microshoots) may be achieved with a Yadollahi, Arab and Shojaeyan (YAS) medium containing 38.2 mM of ${\text{NO}}_{3}^{-}$, 10.45 mM of ${\text{NH}}_{4}^{+}$, 27.35 mM of K^+^, 4.95 mM of Ca^2+^, 1.1 mM of Mg^2+^, 4.5 mM of ${\text{SO}}_{4}^{2-}$, 1.2 mM of ${\text{PO}}_{4}^{2-}$, and 0.48 mM of Cl^−^. It is noteworthy that the YAS medium consists of the average of optimal ions concentrations resulting from ANN-GA for producing optimal microshoots with optimal length. Finally, the YAS medium was compared with other media, such as MS, EM, WPM, and QL, that are commonly used for *Prunus* micropropagation.

**Table 5:** **Optimization analysis on artificial neural network (ANN) model to reach maximum proliferation rate, shoot length, and quality index and minimum callus rate G × N15 prunus rootstock**.

**Item**	**Input variable [Ion concentrations (mM)]**								**Predicted output variable at optimal point**
	$NO_{3}^{-}$	$NH_{4}^{+}$	**K^+^**	**Ca^2+^**	**Mg^2+^**	$SO_{4}^{2-}$	$PO_{4}^{2-}$	**Cl^−^**	
**G × N15**									
Proliferation rate	27.5	14	25.9	5	0.7	4.7	1.1	0.96	12
Shoot length	48.9	6.9	28.8	4.9	1.5	4.3	1.3	0	2.63
Callus rate	28.2	17.6	16.3	4.5	1.4	2.6	1.2	0.07	0.03
Quality index	13.3	5.8	28.2	4.5	0.89	7.6	0.9	1	4.95

## Discussion

One of the major obstacles to the micropropagation of *Prunus* rootstocks up to now has been the lack of a suitable tissue culture medium (Ruzic and Vujovic, 2008). Optimizing the culture medium is one of the most important ways to effect a successful *in vitro* micropropagation. The majority of *Prunus* micropropagation studies of *Prunus* rootstocks have used a single mineral nutrient formulation to determine the effects of various PGRs (Andreu and Marín, 2005; Ruzic and Vujovic, 2008). Unfortunately, because of the complexity of interactions between medium constituents, it is very difficult to determine the optimum levels of minerals and organic compounds for a culture medium (Nas et al., 2013). The wide application of MS, WPM, and QL media as standard growth media for all *Prunus* rootstocks, for the purpose of micropropagation and as a source of leaf explants for transformation and regeneration, has given the false impression that these rootstocks would be difficult to culture if they do not grow well on MS, WPM, and QL media. Previous studies with diverse *Prunus* genotypes confirmed that the so-called standard media require an optimization of nutrients for the successful micropropagation of the unique rootstock or cultivar of *Prunus* (Nas et al., 2013; Nezami Alanagh et al., 2014). Our initial studies showed that the NH_4_${\text{NO}}_{3}^{-}$ and ${\text{KNO}}_{3}^{+}$ components of the MS medium were inadequate for optimal growth and multiplication of G × N15 rootstock (Arab et al., unpublished data). Our earlier studies also showed that replacing Ca(NO_3_)_2_ with CaCl_2_ can improve *in vitro* multiplication of G × N15 (Arab et al., unpublished data). Moreover, *Prunus* rootstocks come in a wide variety of genotypes, with varying nutritional requirements, which makes the medium optimization process more complex and difficult (Espinosa et al., 2006; Canli and Tian, 2008; Ruzic and Vujovic, 2008; Nas et al., 2013; Nezami Alanagh et al., 2014). There are many possible approaches for optimizing the culture medium for plant tissue culture, but there is not a universal approach that can be used either to develop or to modify a micropropagation medium for a large number of species. As a result, time-consuming and complex factorial designs have been applied to optimize the culture medium (Murashige and Skoog, 1962; Niedz and Evens, 2007; Petri and Scorza, 2010). Due to the difficulties involved in developing new formulations, medium development has typically involved comparisons of established media to find one that provides adequate growth and development (Bell et al., 2002), but some researchers have suggested that the levels of medium components can be optimized based on composition of growing whole plants or developing tissues or raw kernels (Staikidou et al., 2006; Ashrafi et al., 2010; Nas et al., 2013). It is likely that more than one medium will be needed for optimum growth of *Prunus* species and cultivars because when MS, EM, and QL media were used for the *in vitro* culture of G × N15, abnormal growth, hyperhydricity, necrosis and discoloration were observed at the multiplication stage, whereas on WPM medium, most shoots were healthy but grew much more slowly (Arab, unpublished data). In order to design an optimized culture media, application a reliable mathematical modeling and optimization method is necessary to reach optimal growth and efficiency (Nezami Alanagh et al., 2014; Jamshidi et al., 2016). Previous studies have used different statistical software to design new and efficient medium culture on *in vitro* condition (Gago et al., 2010a,b; Gallego et al., 2011; Nezami Alanagh et al., 2014; Jamshidi et al., 2016). Response surface method (RSM) has been repeatedly used to optimization of new *in vitro* culture media for pear genotypes (Wada et al., 2013; Jamshidi et al., 2016). Previous investigations reported that ANN-GA models had a significantly higher accuracy of prediction than RSM and stepwise regression models (Ahmadi and Golian, 2011; Sedghi et al., 2012; Jamshidi et al., 2016). Moghri et al. (2015) and Jamshidi et al. (2016) indicated that RSM and regression models alone are not reliable for approximation of non-polynomial or non-linear variables. Recently, it has been reported that the ANN strategy can be an alternative to the traditional statistical methodology, and reduce this long process. Using this methodology does not require a highly specialized background in statistics, yet can optimize a culture process and infer the best conditions (Gago et al., 2010a,b). In this work, we used ANN-GA technology with high learning potential, as a new approach, in order to predict and optimize the combination of mineral nutrient factors (inputs) that influence the growth parameters (outputs) of G × N15 rootstock in the *in vitro* proliferation stage, and to gain new insights into improving the composition of *Prunus* rootstock culture media. In order to predict and optimize the best combination of mineral nutrients, a database of 75 different media was compiled (Tables 1, 2) to model the effect of the ion concentration on the growth parameters using the ANN-GA technique according to Ahmadi and Golian (2011). ANN-GA allows the development of statistical and significant mathematical models characterized by high squared correlation coefficients between predicted and experimental values for all training data (Table 6). There are various methods available for evaluating model performance, including numerical indicators (Table 6) and graphical representations (Figure 2; Shao et al., 2006). As can be seen in Table 6 for each output, the training set *R*^2^ and the test set *R*^2^ obtained were far higher than 75.0% and lower than 96%. *R*^2^ values within these limits have been established as being indicative of good performance, and not overtrained and highly predictable ANN models (Shao et al., 2006). Furthermore, the trained ANN models of outputs yielded balanced statistics for both training and testing data sets (Table 6). This result suggests that overlearning had not occurred during the training process and that the developed models were sufficiently generalizable to successfully treat a previously unseen data set (Lou and Nakai, 2001). Overall, the statistics (Table 6) revealed that the ANN-GA based models could efficiently fit data gained from our experiments on the responses of G × N15 microshoots during *in vitro* multiplication to modified mineral nutrients MS medium. However, one limitation of the ANN modeling technique is that it uses a “black box” approach, which does not allow one to obtain insight into the internal workings of the model or information for evaluating the interactions of the inputs (Dayhoff and DeLeo, 2001). The VSR obtained for the outputs of the models demonstrated that ${\text{NO}}_{3}^{-}$ and ${\text{NH}}_{4}^{+}$ play key roles for all of the growth parameters studied (Tables 4, 5), as they are major sources of nitrogen for G × N15 rootstock micropropagation. The importance of the concentrations of ${\text{NO}}_{3}^{-}$ and ${\text{NH}}_{4}^{+}$ and of the ratio of these concentrations has been widely described (Nowak et al., 2007; Damiano et al., 2009; Ivanova and Van Staden, 2009; Shirdel et al., 2011). The results of this study are in concurrence with those by previous authors that showed that nitrate and ammonium as mineral nutrients play an important role in *in vitro* multiplication of *Prunus* rootstock (Ramage and Williams, 2002; Niedz and Evens, 2007; Nowak et al., 2007; Ivanova and Van Staden, 2009). Many reports on the macro element requirements of *Prunus* sp. in the multiplication stage have predicted total ${\text{NO}}_{3}^{-}$ and ${\text{NH}}_{4}^{+}$ requirements in the ranges of 9.71 mM (Gago et al., 2011) to 12.12 mM (Nezami Alanagh et al., 2014) and 5 mM (Gago et al., 2011) to 10.30 mM (Nezami Alanagh et al., 2014), respectively, for live performance, whereas the ${\text{NO}}_{3}^{-}$ and ${\text{NH}}_{4}^{+}$ contents of experimental diets have ranged from 0.25 mM to 39.41 mM and 0 to 20.61 mM, respectively. The different published values may be due to the different genetics, environmental conditions and dietary factors involved when conducting the experiments (Nezami Alanagh et al., 2014). However, the macro element content of medium is one of the most important determinants of the responses to *in vitro* growth and multiplication. The results obtained from the current study indicated that a proper ratio of ${\text{NO}}_{3}^{-}$ to Ca^2+^ and K^+^ is necessary to obtain the best results. Similarly, Nezami Alanagh et al. (2014) suggested that the GF677 responses to nutrient may vary with the macro level of the element in the culture medium. Analyses of their ANN models for total shoots, healthy shoots and number of nodes from a compiled data set pinpointed the key role of ${\text{NO}}_{3}^{-}$ and its ionic complexes (${\text{NO}}_{3}^{-}$ × Ca^2+^, ${\text{NO}}_{3}^{-}$ × Ca^2+^ × K^+^) on all growth parameters measured. Ca^2+^, as an essential plant nutrient, has many roles in plants such as participating in metabolic processes involving the uptake of other nutrients, promoting the proper elongation of plant cells and strengthening the cell wall structure. Application of this ion at high concentrations in culture media caused shoot necrosis (Nezami Alanagh et al., 2014). In contrast to the results of Nezami Alanagh et al. (2014) our use of the ANN-GA model showed that Ca^2+^ should be at a mid-level concentration (higher than the ~5.0 mM value in MS medium; Table 5) and in a mixture with a high concentration of ${\text{NO}}_{3}^{-}$ (27.5–48.9 mM; Table 5) to maximize the number and lengths of new microshoots (Table 3). Our results showed that to obtain the optimum productivity, the culture medium must be supplemented with a higher concentration (i.e., 25 mM) of K^+^ than the approximate 20 mM concentration of K^+^ that is found in well-established media such as MS or QL. Our results are in contrast with those of Nezami Alanagh et al. (2014) on GF677 that suggested K^+^ at any concentration promotes healthy shoots and high bud number. Our results also suggest that ${\text{PO}}_{4}^{2-}$ has a significant effect on shoot length (Table 5). We also investigated Mg^2+^, which is an indispensable mineral for plant growth and plays a part in many physiological processes (Fontes et al., 1999). The findings of this study showed that relatively low concentrations of Mg^2+^ in the medium resulted in maximum productivity. In contrast, Nezami Alanagh et al. (2014) reported that, compared to MS medium, using a higher concentration of Mg^2+^ (~2.14 mM) in the nutrient medium was required to achieve maximum production.

**Table 6:** **Statistics and information on artificial neural network models for number of micro-shoots, height, callus weight, and quality index of G × N15 plantlet during ***in vitro*** multiplication (training vs. testing values)**.

**Item**	**Proliferation rate**		**Shoot length**		**Callus rate**		**Quality index**	
	**Training**	**Testing**	**Training**	**Testing**	**Training**	**Testing**	**Training**	**Testing**
*R* Square	0.91	0.87	0.95	0.92	0.92	0.87	0.82	0.75
RMSE	0.63	0.77	0.10	0.13	0.013	0.017	0.24	0.30

Many researchers have reported that ${\text{NH}}_{4}^{+}$ plays a key role in *in vitro* culture of *Prunus* rootstock, but at high concentrations, it resulted in hyperhydricity and abnormal growth (Nowak et al., 2007; Ivanova and Van Staden, 2009; Shirdel et al., 2011; Yu et al., 2011; Nezami Alanagh et al., 2014). The results of previous studies showed that the NH_4_${\text{NO}}_{3}^{-}$ component of the MS medium was inadequate for optimal growth and multiplication of *Prunus* sp., leading to hyperhydricity and low quality of plantlets (Nowak et al., 2007; Yu et al., 2011; Arab et al. unpublished data). The ANN-GA model predicted the best results for growth parameters for media including a low amount of ammonium ion (lower than MS; Table 5). Our observation is in agreement with previous findings in which applications of other well-established media, such as WPM and/or QL, containing a reduced proportion of ammonium appeared to have effected efficient *Prunus* micropropagation (Pérez-Tornero et al., 2000). That the reduced amount of ammonium was the causative agent of this improved efficiency is supported by noting that a high dose of ammonium ions (vs. nitrate ions) has been shown to have a negative impact on some growth parameters during *Prunus* rootstock micropropagation (Ivanova and Van Staden, 2009; Radmann et al., 2009; Yu et al., 2011). In our study, plantlets growing on medium containing a high level of ammonium showed hyperhydricity, suggesting that the impact of this factor on the efficiency of micropropagation and quality of plantlets can also be related to additional factors such as the ${\text{NH}}_{4}^{+}$/${\text{NO}}_{3}^{-}$ ratio and the presence of cytokinins, which regulate plant growth (Bosela and Michler, 2008; Shirdel et al., 2011). The ANN-GA model was able to predict and maximize the quantity and average length of microshoots by a combination of decreasing ${\text{NH}}_{4}^{+}$ from a high to low concentration and setting the ${\text{NO}}_{3}^{-}$ concentration at a mid-high level (Table 5). Moreover, by increasing the ${\text{NH}}_{4}^{+}$ concentration, the model predicted a large increase in the number of new microshoots, but also predicted that non-healthy shoots would be obtained. The ANN-GA model predicted that reducing the amount of ${\text{NH}}_{4}^{+}$ in the medium from 20.62 to 5.8 mM would not change microshoots number and height, but would, importantly, increase plantlet quality (Tables 4, 5).

The potential multiplication rates are determined by productivity (number of new microshoots × length of microshoots). Therefore, A superior medium culture should provide a higher mean number of new microshoots per cultured explant, produce longer shoots and be more productive than any commonly used “standard” media. Earlier, for GF677, WPM was found to have a better effect on shoot proliferation rate than either the MS or QL medium, and an explanation given for this was the reduced nitrogen content in WPM (Andreu and Marín, 2005). In addition, other researchers have reported MS, EM, and QL media to be more efficient than other media for *in vitro* proliferation of *Prunus* rootstock (Andreu and Marín, 2005; Radmann et al., 2009). These findings led us to utilize YAS (MS-modified) medium in this study and to compare its effects with those of MS, EM, QL, and WPM media. However, the optimized culture medium YAS was superior to MS, EM, QL, and WPM with respect to the three growth parameters: Shoot length, shoot number per cultured explant and productivity. Nas et al. (2013) reported that productivity may be a helpful growth parameter to consider for selecting the best medium. Higher productivity indicates a high number of long shoots that contain more axillary buds. As the result of being the only medium that provided a high number of long shoots for G × N15, YAS was by far the most productive medium. The differences between productivities observed on different media clearly showed that YAS was the most suitable medium for G × N15 rootstock multiplication. The findings of the present study that YAS medium was more effective compared to MS, EM, QL, and WPM media may be explained by the presence of very high doses of nitrogen in full-strength MS medium, approximately two- to four-fold higher than in QL and WPM. Also, there are differences in the micronutrient compositions of YAS, MS, EM, QL, and WPM media, which might cause differences in the shoot proliferation rates of different species (Andreu and Marín, 2005).

Many researchers of *in vitro* propagation of *Prunus* rootstock have used MS or modifications of this medium, but an increasing number have suggested that concentrations of the inorganic nutrients in the MS medium are inadequate (Pérez-Tornero et al., 2000). Our findings are consistent with the results reported by Nowak et al. (2007), Mansseri-Lamrioui et al. (2009) and Petri and Scorza (2010), which suggested that other well-known media such as WPM and/or QL with lower ammonium concentrations or modifications in mineral composition, mainly reducing nitrate and/or ammonium concentration, of MS have been made in an attempt to optimize *Prunus* micropropagation. The aim of our study was to establish a new approach for prediction-optimization of a new medium formulation as well as to assess the efficiency of this approach by comparing the new formulated medium with media commonly used in *in vitro* multiplication of *Prunus* rootstocks. In the present study, a technique combining artificial neural networks and genetic algorithms (ANN-GA) was applied to an *in vitro* proliferation of the G × N15 rootstock experiment data set. Compared to statistical analysis, ANN-GA more accurately identified interaction effects. Moreover, ANN-GA is less time consuming than statistical analysis, and is particularly helpful when the number of experiments is large. Finally, ANN-GA technology allowed us to determine the optimal combination of factors for achieving the most suitable results for the parameters studied (Tables 4, 5). In addition, comparing the new YAS medium formulated by ANN-GA technology (Tables 4, 5) with standard MS, EM, WPM, and QL growth media for all *Prunus* rootstock showed the YAS medium to be superior (Table 3). ANN-GA should therefore be considered for use as a powerful tool in determining medium formulations, as well as for other areas in plant tissue culture, so that the development of a new nutrient medium can be performed rapidly and efficiently with an increase of productivity, consistency, and quality. According to the results of the current study, application of the ANN-GA technique as a new approach could be extremely useful for designing new and effective optimized culture media in plant tissue culture.

## Author contributions

MA: Designing and performing the experiments, summing up, and writing. AY: Designing and leading. AS: Designing and leading. HA: Statistical analyzing and writing.

### Conflict of interest statement

The authors declare that the research was conducted in the absence of any commercial or financial relationships that could be construed as a potential conflict of interest.

## Abbreviations

WPM: Woody Plant Medium (Lloyd and McCown 1981)
MS: Murashige and Skoog medium (1962)
QL: Quoirin and Lepoivre medium (1977)
YAS, Yadollahi: Arab and Shojaeiyan mediun
EM: Specific Media
BAP: 6-benzyle amino purine
IBA: Indole-3-butyric acid
ANN: Artificial neural network
GA: Genetic algorithm
VSR: Variable sensitivity ratio
PGRs: Plant Growth Regulator.
